# Unequal gains: evaluating Alabama’s infant mortality reduction initiative by race and marital status

**DOI:** 10.1186/s12889-025-25669-7

**Published:** 2025-11-27

**Authors:** Natalie Malak, Holly Horan, David L. Albright

**Affiliations:** 1 Department of Economics , University of Alabama in Huntsville, Huntsville, AL USA; 2 Department of Obstetrics & Gynecology, University of Alabama at Birmingham, Birmingham, AL USA; 3 Department of Political Science, University of Alabama, Tuscaloosa, AL USA

**Keywords:** Infant Mortality, Racial Disparities, Marital Status, Maternal and Child Health, Difference-in-Differences, Synthetic Control Method, Social Determinants of Health

## Abstract

**Background:**

Infant mortality remains a critical public health concern in the United States, with disparities by race and maternal marital status. In response, Alabama implemented a pilot initiative in 2019 to reduce the state’s high infant mortality rate (IMR). This study evaluates the program’s effectiveness in mitigating IMR disparities across race and marital status.

**Methods:**

We utilized linked birth and infant death certificate data from 2014 to 2023 to assess program impact. A difference-in-differences (DiD) design and synthetic control approach were applied to a sample of over 400,000 births, comparing two pilot counties (Montgomery and Macon) to the rest of Alabama. Outcomes were stratified by maternal race (Black, White) and marital status (married, single).

**Results:**

Causal evidence from both the DiD and synthetic control analyses indicates that the program reduced IMR among White married mothers (–0.0016; –39.8%, *p* = 0.001). In contrast, Black married mothers experienced an increase in IMR (+ 0.0043; + 54.7%, *p* = 0.014) that was not statistically robust in DiD but confirmed by the synthetic control method, suggesting a likely causal adverse effect. No significant changes in IMR were detected for Black single (*p* = 0.862) or White single mothers (*p* = 0.777).

**Conclusions:**

The pilot program produced improvements only for White married mothers. Disaggregated analysis revealed the necessity of jointly considering race and marital status to understand and address infant health inequities.

**Public health implications:**

Efforts to reduce IMR disparities must integrate both racial and social determinants—such as marital status. Resource allocation and program design should combine clinical services with broader social supports and prioritize the most vulnerable populations to effectively close persistent gaps in IMR.

**Supplementary Information:**

The online version contains supplementary material available at 10.1186/s12889-025-25669-7.

## Introduction

Infant mortality, defined as the death of an infant before reaching their first birthday, remains a critical indicator of national health and wellbeing. In 2020, the infant mortality rate (IMR) in the United States (US) was 5.42 deaths per 1,000 live births, marking the lowest recorded rate in US history [16]. Despite this progress, the US continues to report the highest IMR among high-resource nations, even with the highest per capita spending on maternal and infant healthcare [21].

The principal causes of infant mortality in the US include congenital malformations, preterm birth and low birth weight, sudden infant death syndrome (SIDS), unintentional injuries, and maternal complications. A complex array of determinants—spanning maternal health conditions, public health practices, healthcare infrastructure, and broader socioeconomic and political factors—contribute to the national IMR [17]. Persistent racial disparities continue to characterize US infant mortality, with Black infants experiencing mortality rates more than twice those of White infants (10.9 vs. 4.5 deaths per 1,000 live births in 2023) [30]. These disparities have been repeatedly documented [22] and are rooted in longstanding inequities [14].

Racial and geographic disparities provide critical entry points for targeted quality improvement strategies to reduce preventable infant deaths. State-level IMRs in 2020 ranged from 3.92 in California to 8.12 in Mississippi [16]. Alabama, a southeastern state, has consistently reported one of the highest IMRs nationally, currently at 7.8 (Alabama Department of Public Health, 2024)[5]. The state also demonstrates significant racial disparities, with an IMR of 13.2 for Black infants and 5.7 for White infants—both of which have worsened since 2020 [5].

In Alabama in 2023, approximately 30% of infants were born to Black mothers, yet this group accounted for 48% of infant deaths.^1^ Marital status further shapes disparities in IMR. Although 60% of Alabama mothers are married, only 46% of infant deaths occur within this group. Notably, 77% of Black mothers in the state are unmarried, compounding the demographic factors associated with elevated risk for infant mortality. This study seeks to disaggregate IMR data not only by race but by the combination of race and marital status.

Despite a persistently high IMR, Alabama has made modest progress over the past decade, attributable to targeted public health efforts, including expanded access to obstetrical care in rural areas [38]. In 2017, Governor Kay Ivey convened the Children’s Cabinet, composed of representatives from six state agencies, to develop a pilot intervention under the State of Alabama Infant Mortality Reduction Initiative. This pilot focused on: (1) increasing access to care, (2) implementing evidence-based population care, and (3) addressing social determinants of health [11]. The program aimed to reduce IMR by 20% over five years, targeting three counties—Montgomery, Macon, and Russell^2^—with interventions including nurse home visits, safe sleep education, and breastfeeding awareness, as well as expanded access to the Well Woman program.^3^

We evaluate the pilot’s impact on IMR and severe infant health outcomes using a novel combination of difference-in-differences (DiD) and synthetic control methods. We further examine how the initiative's effects vary across intersecting demographic groups defined by race and marital status, using confidential linked birth and infant death certificate data from the Alabama Department of Public Health. This study makes a novel contribution to the maternal and child health literature by demonstrating how a state-level infant mortality reduction initiative produced starkly divergent effects once outcomes were disaggregated by both race and marital status.

Previous evaluations of U.S. interventions—such as Medicaid expansion, the Special Supplemental Nutrition Program for Women, Infants, and Children (WIC), and nurse home visiting programs—have generally reported modest reductions in infant mortality, but often without examining subgroup heterogeneity beyond race or income separately [8, 29]. Similarly, state-level reforms like Medicaid postpartum extension have been shown to improve maternal and infant health overall, yet persistent racial disparities remain [23]. International evidence from Canada and Scandinavia underscores that universal maternal care initiatives can reduce national infant mortality rates, but significant disparities persist along socioeconomic and racial lines [20, 25].^4^

Alabama’s pilot program lowered IMR among White married mothers while unintentionally worsening outcomes for Black married mothers. Our findings expand upon prior work by showing that interventions may exacerbate disparities rather than close them, especially when interventions are not designed with vulnerable groups in mind. This investigation underscores the critical need for public health strategies to adopt approaches that integrate race, marital status, and community context into their design and evaluation frameworks.

## Data

### Natality data

This study utilizes linked birth and infant death records from the Alabama Department of Public Health spanning the years 2014 to 2023.^5^ These data offer detailed, individual-level information, enabling precise identification of infant deaths and in-depth analysis of associated birth characteristics. In addition to infant mortality, we examine a composite outcome of severe infant health defined as any of the following: gestational age less than 26 weeks, birth weight below 750 g, or a five-minute APGAR score less than 5.^6^ This outcome serves to detect significant clinical risk, even in cases where mortality is averted.

Explanatory variables include maternal demographic indicators (race, education, age, marital status)^7^ and pregnancy-specific characteristics such as smoking during pregnancy, number of prenatal visits, and a clinical pregnancy risk variable (including diagnoses of diabetes, gestational diabetes, chronic hypertension, gestational hypertension, or pre-eclampsia).^8^ Infant characteristics include birth order (first birth), singleton versus multiple birth, sex, and day of week of birth—an important factor given prior research linking weekend births to poorer outcomes [33].

Additional control variables capture breastfeeding initiation, participation in the Special Supplemental Nutrition Program for Women, Infants, and Children (WIC)**,**^9^ and source of payment for delivery (Medicaid, private insurance, or other). Given that insurance coverage is correlated with maternal risk and infant outcomes, this variable allows for more nuanced estimates of health equity effects [23, 29].

### Aggregate-level controls

We incorporate county-level socioeconomic indicators known to influence infant health outcomes. These include annual unemployment rates from the U.S. Bureau of Labor Statistics, and median household income data from the U.S. Census Bureau. For example, Dehejia and Lleras-Muney [15] find evidence that economic conditions, as proxied by unemployment rates, affect birth outcomes. We also control for COVID-19 case rates per 100,000 population by county to adjust for potential pandemic-related effects.

Recognizing the geographic and health service variation across the state, we also include indicators for urban versus rural county classification and whether rural counties contain hospitals with labor and delivery units.^10^ These structural differences are critical in understanding both access to care and outcomes. Descriptive statistics stratified by policy period and treatment status are presented in Table 1. T-tests were run on all the pre-policy means with no statistically significant differences.

**Table 1:** Summary Statistics

	Pilot Counties (Treatment)		Remaining Alabama Counties (Control)	
	Pre- Pilot Results	During Pilot results	Pre- Pilot Results	During Pilot results
Outcome Variables				
Infant Mortality	0.009	0.009	0.008	0.007
Severe Infant Health	0.018	0.012	0.012	0.011
Demographic Characteristics:				
Black Mother	0.654	0.656	0.289	0.273
Black Father	0.436	0.438	0.208	0.209
White Mother	0.235	0.200	0.618	0.609
White Father	0.210	0.174	0.529	0.525
Hispanic Mother	0.080	0.119	0.074	0.101
Hispanic Father	0.325	0.365	0.246	0.251
Mother High School Degree	0.296	0.349	0.312	0.331
Mother Some College	0.268	0.247	0.300	0.280
Mother Bachelor’s Degree or Higher	0.241	0.221	0.236	0.256
Father High School Degree	0.345	0.386	0.370	0.394
Father Some College	0.219	0.201	0.256	0.234
Father Bachelor’s Degree or Higher	0.251	0.219	0.232	0.241
Teen Mother	0.083	0.076	0.076	0.063
Forty and older Mother	0.020	0.021	0.017	0.022
Teen Father	0.022	0.021	0.023	0.022
Forty and older Father	0.324	0.338	0.244	0.236
Married	0.424	0.376	0.593	0.575
Pregnancy Characteristics:				
Infant Boy	0.517	0.511	0.511	0.510
Number of Prenatal Visits	10.0	9.72	10.7	10.3
Singleton Birth	0.958	0.963	0.964	0.965
Attending Physician	0.904	0.871	0.959	0.913
Medicaid	0.635	0.554	0.509	0.474
Private Insurance	0.303	0.375	0.448	0.481
Smoked during Pregnancy	0.050	0.031	0.105	0.062
First Birth	0.318	0.306	0.332	0.323
Clinically High-Risk Pregnancy	0.150	0.207	0.148	0.223
Initiated Breastfeeding	0.567	0.608	0.672	0.735
Obesity	0.349	0.396	0.312	0.362
Part of WIC Program	0.607	0.507	0.512	0.413
STD Present in Mother	0.031	0.044	0.029	0.034
Abnormal Conditions at Birth	0.100	0.121	0.090	0.115
C-Section Delivery	0.375	0.390	0.348	0.344
County Characteristics:				
Median Household Income	45,109	53,035	47,824	58,598
County Unemployment Rate	5.351	4.496	5.347	3.494
Covid Cases per 100 K	-	13,960	-	14,017
No Labor & Delivery Unit	0.045	0.053	0.070	0.070
Number of Observations	17,815	14,575	287,170	241,530

Severe infant health refers to an infant with either < 750 g, < 26 weeks, or < 5 Apgar score. Clinically high-risk refers to a mother with chronic or gestational diabetes, chronic or gestational hypertension, or eclampsia

## Methods

We first estimate standard difference-in-differences (DiD) models, and second, use synthetic control methods to evaluate the effect of the pilot program on infant health outcomes. DiD models rely on choosing an appropriate comparison group. In this instance, all other counties in Alabama, except Choctaw and the treated counties (Montgomery, Macon, and Russell), will act as our comparison group.^11^ Both Russell County and Choctaw County are excluded from our analysis because they are border counties with 88 and 86 percent of their births occurring over state lines, respectively.^12^ Since a large number of parents deliver in the nearby out-of-state hospital we are unable to see most births in those counties because we are given access only to births inside the state of Alabama.^13^

### Difference-in-differences models

We employ a standard difference-in-differences (DiD) approach with county, year, and month fixed effects to estimate the impact of the pilot program on infant health outcomes.^14^ Our main specification is as follows:

1 $$\begin{aligned} {A}_{ict}&={\beta }_{0}+ {\beta }_{1}{(Treatment}_{c}*{Policy}_{t})+ {\beta }_{2}{X}_{ict}+{\beta }_{3}{P}_{ict} \\ & \quad + {\beta }_{4}{S}_{ct}+ {\beta }_{5}COUNTY+{\beta }_{6}YEAR \\ & \quad+ {\beta }_{7}MONTH+ {\varepsilon }_{ict}\end{aligned}$$

where, ${A}_{ict}$ represents infant mortality or severe infant health for individual *i* in county *c* and year* t*.^15^ The key variable of interest is the interaction term ${Treatment}_{c}*{Policy}_{t}$​, identifying treated counties (Montgomery and Macon) during the pilot period (2019–2023).^16^${X}_{ict}$ and ${P}_{ict}$ represent maternal demographics and pregnancy characteristics, while ${S}_{ct}$​ includes county-level controls for unemployment, income, and COVID-19 case rates.

Given we have very few treated units it’s especially important to cluster our standard errors at the county level [9]. The DiD framework assumes parallel pre-trends between treated and control counties; Appendix Figure A1 provides evidence supporting this assumption.

We estimate models by race and marital status, focusing on Black single, White single, Black married, and White married mothers. As a robustness check we also estimate a “traditional” DiD, where there are no year and county fixed effects, so we include a treatment and policy period fixed effect. Results of the “traditional” DiD match our main specification and can be found in Appendix Table 2.

### Synthetic control models

To validate and supplement our DiD estimates, we apply synthetic control methods [1–4]. This approach constructs a counterfactual outcome trajectory by weighting untreated counties (the donor pool) to closely match treated counties' pre-intervention trends. When comparison units are not sufficiently like the treated unit the variation in outcomes may be due to the difference in their characteristics, rather than the policy intervention or event of interest [18, 19, 24].

In the absence of randomization, there is no one county that acts as a perfect comparison for another. The main idea behind the synthetic control approach is that a weighted combination of counties will provide a better counterfactual than any one county alone. Suppose that there are *J* counties, where *i* = *1* refers to Montgomery and Macon, the treated group. Borrowing from the statistical matching literature, we refer to the set of potential controls as the ‘donor pool’, that is, all the counties in Alabama that are not part of the pilot program. The synthetic control method identifies a linear combination of the other *i* = *2* to *J* counties with weights, *W* = *(w*_*2*_*,….w*_*J*_*)* such that *w*_*i*_ ≥ *0* and *w*_*2*_ + *….* + *w*_*J*_ = *1*, that best reproduces the treated group during the pre-pilot period, in terms of a list of observed controls (*Z*) that potentially affect infant mortality.^17^ The identification of the effect of the pilot program is achieved by comparing the post-reform *observed* infant mortality in the treated group, *Y*_*1*_, and its synthetic equivalent, *Y*_*S*_ = Σ*w*_*i*_*Y*_*i*_, *i* = *2* to *J.* The estimated treatment effect at time *t* > T_0_, where T_0_ is the last pre-treatment period, is:

2 $${\alpha }_{1t}={Y}_{1t}-\Sigma {w}_{i}{Y}_{it}$$

where *Y*_*1t*_ is the observed outcome for the treated group. Synthetic control provides a transparent and data-driven method of adjusting for observable and unobservable confounding when randomization is infeasible.

## Results

### Difference-in-differences

**Fig. 1:**
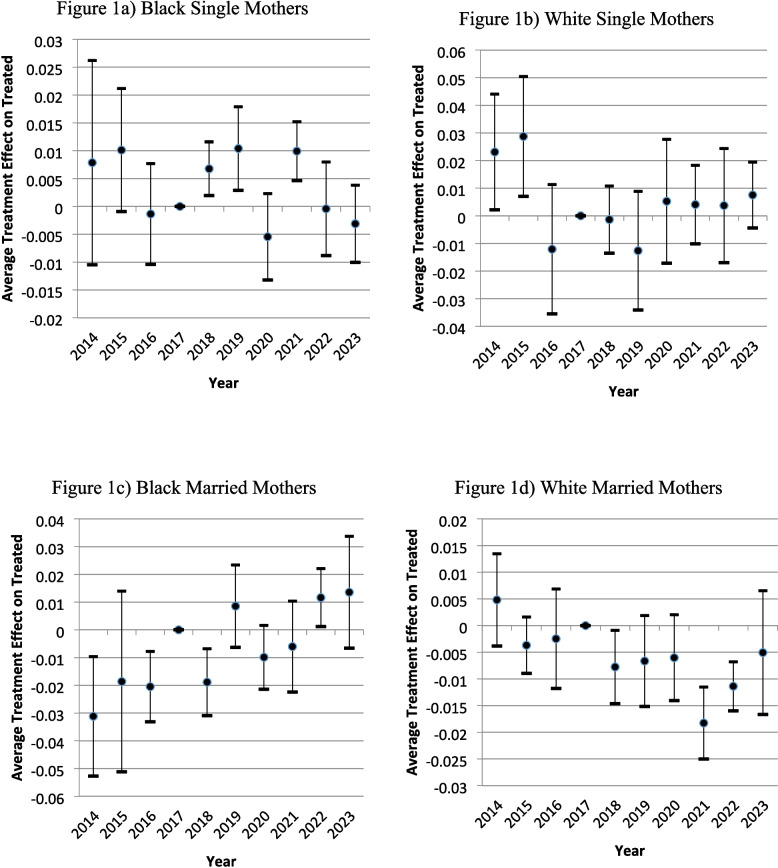
Infant Mortality Rate (IMR) event study

Table 2 reports DiD estimates by combined race and marital status, with Fig. 1 showing the corresponding event studies^18^,^19^,^20^. Among single mothers, neither Black nor White subgroups showed significant changes in IMR, although improvements in severe infant health were observed. White married mothers experienced a statistically significant 0.0016 point decline in IMR—equivalent to a ~ 40% reduction from a pre-policy mean of 4 deaths per 1,000. Conversely, Black married mothers exhibited a statistically significant increase in IMR but were sensitive to the parallel trend assumption. Although magnitudes are large, absolute values remain small and should be interpreted cautiously. Notably, the sample of White married mothers (*N* = 231,215) is approximately six times larger than that of Black married mothers (*N* = 37,700).^21^ We further check the sensitivity of our definition of severe infant health with a combination of different definitions, altering the birthweight, gestational age, and APGAR scores. Appendix Table A5 shows that our results hold even with the variation in our definition of severe infant health.

**Table 2:** Outcomes of pilot program by race & marital status combinations

	Black Single Mothers	White Single Mothers	Black Married Mothers	White Married Mothers
Panel A: Infant Mortality Rate Outcome				
Coefficient${\beta }_{1}$	0.0003	−0.0003	0.0043	−0.0016
Standard Error	0.0015	0.0010	0.0017	0.0005
* P*-value	0.8620	0.7770	0.0140	0.0010
Treatment Pre-Policy Mean	0.0116	0.0115	0.0079	0.0041
Relative Change (%)	2.3	−2.4	54.7	−39.8
Observations	71,230	61,135	37,700	231,215
Panel B: Severe Infant Health Outcome (< 750 g, < 26 weeks, or < 5 Apgar)				
Coefficient${\beta }_{1}$	−0.0079	−0.0076	−0.0018	−0.0027
Standard Error	0.0014	0.0017	0.0025	0.0005
* P*-value	< 0.0010	< 0.0010	0.4580	< 0.0010
Treatment Pre-Policy Mean	0.0237	0.0141	0.0176	0.0055
Relative Change (%)	−33.2	−53.9	−10.4	−48.0
Observations	71,230	61,135	37,700	231,215

Each coefficient is the policy's effect from a separate difference-in-differences model with county fixed effects, year fixed effects, month fixed effects, and all other explanatory variables. Standard errors are clustered at the county level. The relative change is calculated as (coefficient ÷ treatment group’s pre-policy mean) × 100

The divergence between infant mortality and severe infant health outcomes—particularly for Black single mothers—likely reflects differences in what these measures capture. Severe health indicators (very low birthweight, extreme prematurity, low APGAR scores) improved modestly, suggesting that prenatal care and clinical management were somewhat effective. However, these gains did not translate into lower mortality, which also depends on postnatal care, safe sleep, and timely access to pediatric services. Thus, we examine the differences in neonatal (death within 28 days of birth) and postneonatal (death after the first 28 days but within the first year of life) outcomes in Table 3. Here we observe that White married mothers are seeing improvement across both stages, whereas we can pinpoint that Black mothers (regardless of marital status) are seeing the increase in infant mortality in the postneonatal phase, this implies concerns related to postnatal complications, for example, SIDS (see [27]). The pilot did include a safe sleep initiative with digital media campaigns and various streaming platforms. It could be the case that the channels they were using were not effectively reaching Black mothers.

**Table 3:** Outcomes of pilot program by race & marital status- neonatal vs postneonatal

	Black Single Mothers	White Single Mothers	Black Married Mothers	White Married Mothers
Panel A: Neonatal Death Outcome				
Coefficient${\beta }_{1}$	−0.0016	−0.0045	0.0013	−0.0010
Standard Error	0.0013	0.0006	0.0014	0.0004
*P*-value	0.2360	< 0.0010	0.3430	0.0140
Treatment Pre-Policy Mean	0.0062	0.0078	0.0065	0.0023
Relative Change (%)	−25.8	−57.7	20.0	−43.5
Observations	71,230	61,135	37,700	231,215
Panel B: Postneonatal Death Outcome				
Coefficient${\beta }_{1}$	0.0019	0.0041	0.0030	−0.0005
Standard Error	0.0005	0.0009	0.0006	0.0003
* P*-value	< 0.0010	< 0.0010	< 0.0010	0.0750
Treatment Pre-Policy Mean	0.0056	0.0039	0.0015	0.0018
Relative Change (%)	33.9	105.1	200.0	−27.8
Observations	71,230	61,135	37,700	231,215

Each coefficient is the policy's effect from a separate difference-in-differences model with county fixed effects, year fixed effects, month fixed effects, and all other explanatory variables. Standard errors are clustered at the county level. The relative change is calculated as (coefficient ÷ treatment group’s pre-policy mean) × 100. Neonatal are infant deaths that occur within 28 days, all other deaths before the age of one are considered postneonatal deaths

Appendix Table A6 and A7 provide further heterogeneity analysis based on mother’s age and education, respectively. We can see improvements in White mothers under 30 years of age, but Black married mothers over 30 years old are experiencing an increase in infant mortality. These patterns align with theories of unequal access to resources which are discussed in Sect. "Potential mechanisms". Younger and more educated White mothers may have greater access to, and capacity to act on, new health interventions, functioning as early adopters. In contrast, older Black mothers—despite marital status—may face compounded structural barriers and cumulative stress over the life course, which limits their ability to benefit equally from the same interventions. These disparities suggest that age and education amplify existing racial inequities in access to the social, economic, and technological resources that mediate health outcomes.

Another robustness check presented in Appendix Table A8 only uses bordering counties^22^ as controls and yields similar results. Appendix Tables A9 and A10 show multiple robustness checks for infant mortality and severe infant health, respectively. These tables show the beta estimates for Oster’s [32] test when delta equals 1 and −1, illustrating results may be sensitive to omitted variable bias. Panel B removes the Covid-19 Lockdown period from March 2020 to April 2021 from our data. We acknowledge that the pilot program couldn’t fully run as intended during that time period, as nurse home visits had to transition to a virtual format. When excluding these years, our main results still hold. Panel C runs year-month fixed effects instead of separate year and month fixed effects. Results remain consistent. Finally, Panel D and E estimate logit and probit models, and statistically significant results hold.

### Synthetic control

**Fig. 2:**
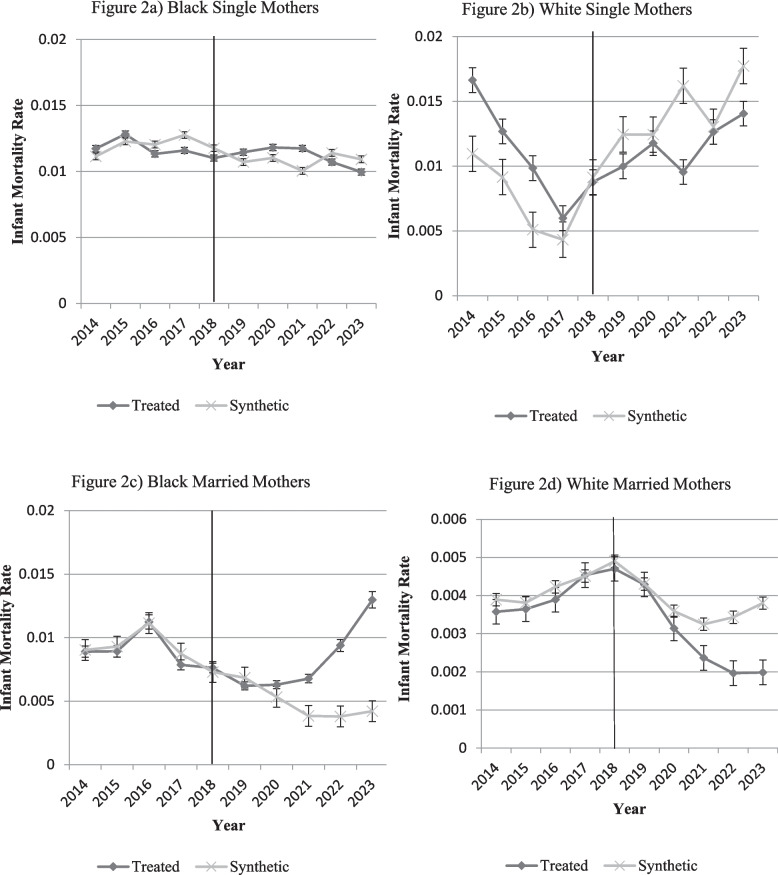
Synthetic control by race and marital status combinations

Our robustness checks, as well as general comments on DiD, always revert to whether the control group is the ‘right’ control group. With synthetic controls, we can construct a more precise control group from the donor pool of available counties to create a “synthetic” treated group. Figure 2 displays the synthetic control method for infant mortality by race and marital status combined. Appendix Table A11 lists which counties, along with their assigned weight, were used to form the “synthetic” treated group for each race/marital status combination. It also notes the Root Mean Squared Prediction Error (RMSPE) for each synthetic control group. Based on the RMSPE the synthetic control model did a better job of matching Black and White married mothers.

The synthetic control graphs in Fig. 2 show that the results in the DiD match closely with the synthetic control method. Figure 2a and b illustrate a lack of difference between the treated and synthetic group for Black single and White single mothers, respectively. Although statistical differences may exist, there is no consistent story unlike the other two groups. Figure 2c and d show a more precise pre-policy match between the treated and synthetic counties with a growing difference after the policy, an increase for Black married mothers and a decrease for White married mothers. The synthetic control annual treatment effects and average treatment effect in Appendix Table A12 confirm the DiD results from Table 2, and show a relatively similar average treatment effect, although we do note the synthetic control estimates are more conservative. Together, both methods consistently highlight improvements only for White married mothers and the synthetic control method illustrates a concerning divergence for Black married mothers.

### Potential mechanisms

The divergent program impacts observed between White married and Black married mothers may be attributable to several mechanisms. First, the bundled intervention components—such as breastfeeding promotion, nurse home visits, and Well Woman care—may have been better aligned with the needs and healthcare access patterns of White married women, who were more likely to have private insurance and stronger provider continuity. In contrast, Black married women often encounter implicit bias, and differential quality of care within healthcare settings, which can undermine program effectiveness despite similar service availability. The Fundamental Cause Theory [26] argues that health disparities persist because groups with more resources (economic, social, cultural) are better positioned to take advantage of new health interventions. This ties with the Diffusion of Innovations Theory by Rogers [36] that suggest that adoption of new health practices spreads unevenly, often starting with more advantaged groups. White married mothers may represent the “early adopters” of these interventions, producing asymmetric benefits.

Moreover, structural disadvantages were especially pronounced in Montgomery County, where Black mothers represented two-thirds of births but nearly four-fifths of infant deaths. This is indicative of community-level inequities that limit the protective benefits of marriage. In fact, marriage itself may confer unequal protective effects across racial groups, as socioeconomic and social capital advantages associated with marriage among White women are not equally realized among Black women. The Weathering Hypothesis (Geronimus, 1992) suggests that socioeconomic disadvantage leads to chronic stress and earlier health deterioration among Black women, especially in reproductive outcomes. The transition of home visits to virtual formats during the COVID-19 pandemic likely compounded these disparities, disproportionately reducing effectiveness for families with higher-risk pregnancies or less reliable digital access [6]. Furthermore, the economic fallout from the pandemic also had unequal impacts by race. Black women were more likely to be employed in jobs without remote work flexibility, paid leave, or childcare support, which may have limited their ability to engage consistently with health interventions [10]. In contrast, White married women were more likely to benefit from greater employment stability, access to remote work, and dual-income household advantages [13]. These structural differences likely affected not only the reach and uptake of the intervention but also the underlying conditions necessary to benefit from it. Collectively, these factors help explain why the initiative improved infant outcomes for White married mothers while widening disparities for their Black married counterparts.

In Appendix Table A13 we examine some potential mechanisms by estimating the effect of the pilot program on delivery payment method (private insurance versus Medicaid), as well as the WIC program. We find the increase in private insurance is met with a decrease in Medicaid, and no change for White married women. Furthermore, there was no change in WIC except for Black single mothers seeing a decrease. We encourage further research to examine whether the lack of improvement for Black single mothers could be related to less access to programs,^23^ such as WIC, as well as the opposing signs for delivery payment method (private insurance versus Medicaid).

## Discussion and public health implications

Our findings suggest that Alabama’s Infant Mortality Reduction Initiative yielded modest benefits, concentrated among White married mothers. The program’s divergent effects likely reflect differences in access to care, alignment of services with health needs, with marriage conferring stronger protective benefits for White than Black families [14, 22, 23, 28]. These differential impacts underscore the need for targeted intervention strategies tailored to subpopulations most at risk, particularly Black and single mothers.

Although we control for county-level economic and healthcare access variables, we cannot account for all contemporaneous local policies or disruptions—such as the COVID-19 pandemic, during which nurse home visits transitioned to virtual formats. The impact of these changes on care quality remains uncertain.

Future evaluations should disaggregate outcomes by individual program components. The bundled nature of services (e.g., Well Woman, nurse visits, breastfeeding and safe sleep campaigns) limits attribution to specific elements. A more rigorous design—incorporating staggered implementation or randomized clinic assignments—could better isolate effective components.

Our analysis also aligns with decades of prior research identifying Black and single mothers as particularly vulnerable to poor infant health outcomes [8, 37]. The demographic disparities observed in Montgomery County over our sample period (2014–2023)—where Black mothers constitute 65% of births but 77% of deaths, and single mothers account for 59% of births but 75% of deaths—reinforce the urgency for tailored intervention.

In sum, the Alabama Infant Mortality Reduction Initiative demonstrates both the promise and the peril of state-level interventions: while White married mothers experienced measurable gains, Black married mothers saw worsened outcomes, underscoring the danger of race-neutral or marital-status-neutral designs. These results highlight the necessity of recognizing how race and marital status jointly shape risk—and of tailoring program delivery to address barriers, cultural contexts, and community needs. We further note, that postneonatal care and outreach should be a priority for Black and single mothers, given the increase in postneonatal mortality compared to White married mothers. Future initiatives should combine clinical services with broader social supports and ensure continuous disaggregated evaluation to prevent unintended disparities and achieve equitable improvements in infant survival. For example, program components like breastfeeding promotion and safe sleep education could be paired with more intensive postneonatal outreach and culturally tailored care delivery for vulnerable mothers. We suggest that future initiatives explicitly address structural barriers—such as unequal access to follow-up pediatric care and persistent bias in healthcare delivery—by integrating broader social supports (e.g., home visiting with transportation assistance, digital access, and stress-reduction interventions).

## Conclusion

This study provides one of the first empirical evaluations of a state-led initiative targeting infant mortality using a combined difference-in-differences and synthetic control framework. While the intervention showed promising outcomes for White married mothers, it failed to deliver improvements for Black or single mothers—groups historically and currently most affected by infant mortality.

While the initiative reduced infant mortality among White married mothers, it coincided with worsening outcomes for Black married mothers. This divergence likely reflects unequal benefits of marriage across racial groups, compounded by inequities in healthcare, community-level disadvantage, and disruptions in service delivery during COVID-19, underscoring the need for more targeted, equity-focused program design.

The findings highlight the necessity of integrating demographic stratification—particularly race and marital status—into the design, implementation, and evaluation of public health programs. Alabama’s experience shows that assuming marriage is universally protective can obscure risk among Black married mothers. Future interventions should prioritize intersectional targeting, culturally tailored care models, and disaggregated evaluation to detect unintended harms early. Pairing clinical services with structural supports such as housing, food security, and transportation assistance and postneonatal outreach is essential to closing persistent gaps in infant survival.

## Supplementary Information

Supplementary Material 1.

## Data Availability

Data can be requested by the Alabama Department of Public Health.

## Footnotes

1 The percentages in this paragraph are coming directly from our data. We have the linked death certificates to all the birth certificates in Alabama. Please see data section for further details.

2 Following a county-level review of infant mortality data from 2014–2016, the Children’s Cabinet identified counties with high rates, from which Montgomery, Macon, and Russell were selected as pilot sites. In this study we only focus on Montgomery and Macon due to a lack of data for Russell County. The majority of Russell residents cross the state border to deliver their newborns, and thus we do not have the birth certificates for these births. Detailed reports on the Infant Mortality Reduction Initiative can be found here: https://www.alabamapublichealth.gov/perinatal/reduction.html

3 Most of the funding was spent on the provision of direct care through nurse home visits and the *Well Woman* program. Specifically, 70% of the first year’s annual $1 million budget was allocated to these two initiatives.

4 Likewise, studies in Belgium and across Europe show that immigrant and marginalized women experience worse perinatal outcomes despite universal coverage, reflecting enduring structural disadvantage [31, 34].

5 In 2014, internal coding of the birth certificate variables changed so there was concern about potential variations in merging birth certificates from prior years. Birth certificates are linked to infant death occurring within the first year of life. Thus, the 2023 birth certificates are linked as far out as December 2024 to death certificates.

6 An APGAR score is a measure from zero to ten that quickly summarizes the health of a newborn baby at 5 min after birth.

7 We also include father’s race, education, and age indicators, although there is a substantial amount of missing data with respect to these variables (specifically 43% for Black single; 29% for White single; 7% for Black married; and 3% for White married). For robustness we re-estimate our main results Table 2 excluding all father’s controls (race, education, and age) with no change to results.

8 Diabetes is a metabolic disease in which the body’s inability to produce any or enough insulin causes elevated levels of glucose in the blood. Gestational diabetes is a clinical condition specific to pregnancy when a woman without a previous history of diabetes develops high blood sugar levels. This condition is typically diagnosed between 24 and 28 weeks of pregnancy. Chronic hypertension is defined as having blood pressure greater than 140/90 mm Hg. Gestational hypertension is the development of new hypertension in a pregnant woman after 20 weeks gestation without the presence of protein in the urine or other signs of pre-eclampsia. Eclampsia is a condition in which one or more convulsions occur in a pregnant woman suffering from high blood pressure and proteinuria, often followed by coma and posing a threat to the health of both mother and baby.

9 This is a program to safeguard the health of low-income women, infants, and children from birth to age 5 who are at risk for malnutrition.

10 This data was graciously provided by Waits et al. [38].

11 For a robustness check we will limit the comparison group to all the surrounding counties among the treated counties.

12 We calculate these percentages by sourcing the national U.S. vital statistics. In the national data we can decipher between the state the birth occurred in versus the state the mother resides in. The next highest county is Sumter county, with 65 percent of births occurring outside the state.

13 The authors feel it is important for policy makers to note that most infant deaths in Russell County are coming from infants born outside the state. This information was provided by the Alabama Department of Public Health.

14 We use linear probability models for ease of interpreting absolute change in IMR.

15 Appendix Table 1 shows summary statistics for the outcome variables by each group.

16 Funding began in October 2018.

17 The observed controls (Z) were age and education indicators of the mother, as well as the pregnancy controls found in Table 1, and Eq. (1). These variables are used to create the best weighted match for the treated units, and how well it fits is the Root Mean Squared Prediction Error (RMSPE).

18 For multiple hypothesis testing in Table 2 we estimated Romano-Wolf [12] adjusted p-values, as well as the False Discovery Rate [7]. All statistically significant results remained so with the Romano-Wolf adjusted p-values, and the False Discovery Rate had a corrected overall critical p-value of 0.079, again implying all statistically significant results remain so.

19 For sensitivity analysis we also re-estimated Table 2 removing one county at a time and the results were not driven by any particular county.

20 Appendix Figure A2 tests the sensitivity of the parallel trend assumption by implementing the Rambachan and Roth [35] sensitivity plots. Based on these plots Black single mothers and especially White married mothers are not sensitive to the change in the trends. Both White single and Black married mothers are sensitive to the parallel trends assumption. This is not surprising given the lower number of observations and lack of parallel trends in Appendix Figure A1.

21 Appendix Table A3 and A4 show the robustness of adding and removing controls to the results of Black married and White married mothers, respectively.

22 Border counties consist of Autauga, Barbour, Bullock, Crenshaw, Elmore, Lee, Lowndes, Pike and Tallapoosa.

23 If funds were reallocated from other programs to support the infant mortality reduction initiative, there is the possibility of unintended effects.

## References

[1] Abadie A, Diamond A, Hainmueller J. Synthetic control methods for comparative case studies: estimating the effect of California’s tobacco control program. J Am Stat Assoc. 2010;105(490):493–505.

[2] Abadie A, Diamond A, Hainmueller J. Synth: an R package for synthetic control methods in comparative case studies. J Stat Softw. 2011;42(13):1–17.

[3] Abadie A, Diamond A, Hainmueller J. Comparative politics and the synthetic control method. Am J Polit Sci. 2015;59(2):495–510.

[4] Abadie A, Gardeazabal J. The economic costs of conflict: a case study of the Basque Country. Am Econ Rev. 2003;93(1):112–32.

[5] Alabama Department of Public Health. Infant Mortality Alabama 2023. Center for Health Statistics; 2024. Available from: https://www.alabamapublichealth.gov/healthstats/assets/infantmortality2023.pdf

[6] Al-Taiar A, Kekeh MA, Ewers S, et al. Virtual home visits during COVID-19 pandemic: mothers’ and home visitors’ perspectives. BMC Pregnancy Childbirth. 2023;23:577. 10.1186/s12884-023-05896-9. 37568102 10.1186/s12884-023-05896-9 PMC10422766

[7] Anderson ML. Multiple inference and gender differences in the effects of early intervention: a reevaluation of the Abecedarian, Perry Preschool, and Early Training projects. J Am Stat Assoc. 2008;103(484):1481–95.

[8] Barkauskas VH. Effectiveness of public health nurse home visits to primiparous mothers and their infants. Am J Public Health. 1983;73(5):573–80. 6837823 10.2105/ajph.73.5.573 PMC1650846

[9] Bertrand M, Duflo E, Mullainathan S. How much should we trust differences-in-differences estimates? Q J Econ. 2004;119(1):249–75.

[10] Byambasuren B, Hegewisch A, Watson Ellerbe R. The intersection of workplace flexibility and exercise by gender, race, and ethnicity. Institute for Women’s Policy Research. 2025. https://iwpr.org/wp-content/uploads/2025/03/The-Intersection-of-Workplace-Flexibility-and-Exercise-by-Gender-Race-and-Ethnicity-report_202523.pdf

[11] Children’s Cabinet. State of Alabama Infant Mortality Reduction Plan. Alabama Department of Public Health; 2018. Available from: https://www.alabamapublichealth.gov/perinatal/assets/alabama-im-reduction-plan.pdf

[12] Clarke D, Romano J, Wolf M. The Romano-Wolf multiple hypothesis correction in Stata. SSRN Electron J. 2020. 10.2139/ssrn.3513680.

[13] Congdon WJ, Jacobs ES, Sawo M. Labor market policies for racial equity. Urban Institute. 2024. https://www.urban.org/sites/default/files/2024-04/Labor_Market_Policies_for_Racial_Equity.pdf

[14] Cooper Owens D, Fett SM. Black maternal and infant health: historical legacies of slavery. Am J Public Health. 2019;109(10):1342–5. 31415204 10.2105/AJPH.2019.305243 PMC6727302

[15] Dehejia R, Lleras-Muney A. Booms, busts, and babies’ health. Q J Econ. 2004;119(3):1091–130.

[16] Ely DM, Driscoll AK. Infant mortality in the United States, 2020: data from the period linked birth/infant death file. Natl Vital Stat Rep. 2022;71(5). 10.15620/cdc:120700 36190428

[17] Ely DM, Driscoll AK, Mathews TJ. Infant mortality by age at death in the United States, 2016. NCHS Data Brief. 2018;(326). 30475688

[18] Geddes B. Paradigms and sand castles: theory building and research design in comparative politics. Ann Arbor: University of Michigan Press; 2003.

[19] George GAL, Bennett A. Case studies and theory development in the social sciences. Cambridge (MA): MIT Press; 2005.

[20] Gissler M, Rahkonen O, Järvelin MR, Hemminki E. Social class differences in health until the age of seven years among the Finnish 1987 birth cohort. BMJ. 1998;316(7132):338–9. 10.1016/s0277-9536(98)00013-6 9672394

[21] Gunja MZ, Gumas ED, Williams RD. U.S. health care from a global perspective, 2022: accelerating spending, worsening outcomes. The Commonwealth Fund; 2023. Available from: 10.26099/8ejy-yc74

[22] Hogue CJ, Vasquez C. Toward a strategic approach for reducing disparities in infant mortality. Am J Public Health. 2002;92(4):552–6. 11919050 10.2105/ajph.92.4.552 PMC1447115

[23] Johnson DL, Carlo WA, Rahman AKMF, Tindal R, Trulove SG, Watt MJ, et al. Health insurance and differences in infant mortality rates in the US. JAMA Netw Open. 2023;6(10):e2337690. 10.1001/jamanetworkopen.2023.37690. 37831450 10.1001/jamanetworkopen.2023.37690 PMC10576209

[24] King G, Keohane RO, Verba S. Designing social inquiry: scientific inference in qualitative research. Princeton (NJ): Princeton University Press; 1994.

[25] Kramer MS, Séguin L, Lydon J, Goulet L. Socio-economic disparities in pregnancy outcome: why do the poor fare so poorly? Paediatr Perinat Epidemiol. 2000;14(3):194–210. 10.1046/j.1365-3016.2000.00266.x. 10949211 10.1046/j.1365-3016.2000.00266.x

[26] Link BG, Phelan J. Social conditions as fundamental causes of disease. J Health Soc Behav. 1995;Spec No:80-94. PMID: 7560851. 7560851

[27] Mathews AA, Joyner BL, Oden RP, Alamo I, Moon RY. Comparison of infant sleep practices in African-American and US Hispanic families: implications for sleep-related infant death. J Immigr Minor Health. 2015;17(3):834–42. 10.1007/s10903-014-0016-9. 24705738 10.1007/s10903-014-0016-9 PMC4185304

[28] McLanahan S, Percheski C. Family structure and the reproduction of inequalities. Annu Rev Sociol. 2008;34:257–76.

[29] Moss NE, Carver K. The effect of WIC and Medicaid on infant mortality in the United States. Am J Public Health. 1998;88(9):1354–61. 9736876 10.2105/ajph.88.9.1354 PMC1509087

[30] National Center for Health Statistics. Infant mortality in the United States, provisional data from the 2023 period linked birth/infant death file. Natl Vital Stat Rep. 2025;74(7). Available from: https://www.cdc.gov/nchs/data/nvsr/nvsr74/nvsr74-07.pdf 10.15620/cdc/174592 PMC12451502 40956638

[31] Norredam M, Nielsen SS, Krasnik A. Migrants’ utilization of somatic healthcare services in Europe—a systematic review. Eur J Public Health. 2010;20(5):555–63. 20040522 10.1093/eurpub/ckp195

[32] Oster E. Unobservable selection and coefficient stability: theory and evidence. J Bus Econ Stat. 2016;34(2):187–204.

[33] Palmer W, Bottle A, Aylin P. Association between day of delivery and obstetric outcomes: observational study. BMJ. 2015;351:h5774. 26602245 10.1136/bmj.h5774 PMC4658392

[34] Racape J, De Spiegelaere M, Alexander S, Dramaix M, Buekens P. Perinatal health inequalities between natives and immigrants in Belgium: role of social disadvantage. Int J Public Health. 2010;55(5):443–9.20401513

[35] Rambachan A, Roth J. A more credible approach to parallel trends. Rev Econ Stud. 2023;90(5):2555-91. 10.1093/restud/rdad018.

[36] Rogers, EM. Diffusion of Innovations. Free Press of Glencoe;1962.

[37] Smiley J, Eyres S, Roberts DE. Maternal and infant health and their associated factors in an inner city population. Am J Public Health. 1972;62(4):476–82. 5020611 10.2105/ajph.62.4.476 PMC1530123

[38] Waits JB, Smith L, Hurst D. Effect of access to obstetrical care in rural Alabama on perinatal, neonatal, and infant outcomes: 2003–2017. Ann Fam Med. 2020;18(5):446–51. 10.1370/afm.2580. 32928761 10.1370/afm.2580 PMC7489970

