# Supplementary material for: Unequal gains: evaluating Alabama’s infant mortality reduction initiative by race and marital status
Source: BMC Public Health. 2025 Nov 27;25:4340. doi: 10.1186/s12889-025-25669-7 (PMC12751583; doi:10.1186/s12889-025-25669-7)
Supplement: Supplementary file 1 — Supplementary Material 1. [file 12889_2025_25669_MOESM1_ESM.docx]

| Appendix Table A1: Outcome Variables by Race and Marital Status | | | | | |
| --- | --- | --- | --- | --- | --- |
|  | Pilot Counties  (Treatment) | | Remaining Alabama Counties (Control) | | |
|  | Pre- Pilot Results | During Pilot Results | Pre- Pilot Results | During Pilot Results |  |
| Black Single Mothers: |  |  |  |  |  |
| Infant Mortality | 0.0118 | 0.0110 | 0.0128 | 0.0124 |  |
| Severe Infant Health | 0.0239 | 0.0147 | 0.0206 | 0.0197 |  |
| N | 9,000 | 7,560 | 61,690 | 50,140 |  |
|  |  |  |  |  |  |
| White Single Mothers: |  |  |  |  |  |
| Infant Mortality | 0.0117 | 0.0102 | 0.0079 | 0.0079 |  |
| Severe Infant Health | 0.0143 | 0.0068 | 0.0108 | 0.0104 |  |
| N | 770 | 590 | 46,465 | 40,260 |  |
|  |  |  |  |  |  |
| Black Married Mothers: |  |  |  |  |  |
| Infant Mortality | 0.0079 | 0.0090 | 0.0117 | 0.0087 |  |
| Severe Infant Health | 0.0177 | 0.0135 | 0.0192 | 0.0166 |  |
| N | 2,655 | 2,000 | 21,170 | 15,800 |  |
|  |  |  |  |  |  |
| White Married Mothers: |  |  |  |  |  |
| Infant Mortality | 0.0041 | 0.0026 | 0.0047 | 0.0040 |  |
| Severe Infant Health | 0.0056 | 0.0043 | 0.0072 | 0.0073 |  |
| N | 3,415 | 2,325 | 130,915 | 106,720 |  |

Notes: Severe infant health refers to an infant with either <750 grams, <26 weeks, or <5 Apgar score. N represents number of observations. T-tests were performed on the pre-pilot means and the differences were not statistically significant.

| Appendix Table A2: Outcomes of Pilot Program- Traditional DiD | | | |  |  |
| --- | --- | --- | --- | --- | --- |
|  | All  Mothers | Black Single Mothers | White Single  Mothers | Black Married  Mothers | White Married Mothers |
| Panel A: Infant Mortality Rate Outcome | | | | | |
| Coefficient $\beta_{1}$ | 0.0016 | 0.0007 | 0.0002 | 0.0046 | -0.0011 |
| Standard Error | 0.0008 | 0.0013 | 0.0007 | 0.0011 | 0.0003 |
| P-value | 0.0490 | 0.5540 | 0.8160 | <0.0010 | 0.0010 |
| Treatment Pre-Policy Mean | 0.0089 | 0.0116 | 0.0115 | 0.0079 | 0.0041 |
| Relative Change (%) | 17.8 | 6.5 | 1.4 | 58.4 | -27.6 |
| Observations | 451,160 | 71,230 | 61,135 | 37,700 | 231,215 |
|  |  |  |  |  |  |
| Panel B: Severe Infant Health Outcome (<750 g, <26 weeks, or <5 Apgar) | | | | | |
| Coefficient $\beta_{1}$ | -0.0046 | -0.0100 | -0.0077 | -0.0019 | -0.0031 |
| Standard Error | 0.0005 | 0.0011 | 0.0013 | 0.0015 | 0.0005 |
| P-value | <0.0010 | <0.0010 | <0.0010 | 0.2270 | <0.0010 |
| Treatment Pre-Policy Mean | 0.0175 | 0.0237 | 0.0141 | 0.0176 | 0.0055 |
| Relative Change (%) | -26.3 | -42.4 | -54.8 | -10.6 | -56.6 |
| Observations | 451,160 | 71,230 | 61,135 | 37,700 | 231,215 |
| Notes: Each coefficient is the policy's effect from a separate difference-in-differences model with no county or year fixed effects, but all other explanatory variables. Standard errors are clustered at the county level. The relative change is calculated as (coefficient ÷ treatment group’s pre-policy mean) × 100. | | | | | |

| Appendix Table A3: Outcomes of Pilot Program on Black Married Mothers | | | | | |
| --- | --- | --- | --- | --- | --- |
| Panel A: Infant Mortality Rate Outcome | | | | | |
| Coefficient $\beta_{1}$ | 0.0011 | 0.0038 | 0.0037 | 0.0042 | 0.0043 |
| Standard Error | 0.0006 | 0.0010 | 0.0010 | 0.0011 | 0.0017 |
| P-value | 0.0810 | <0.0010 | 0.0010 | <0.0010 | 0.0140 |
| Relative Change (%) | 13.9 | 48.2 | 46.3 | 52.5 | 54.7 |
| Observations | 41,620 | 41,620 | 41,620 | 38,515 | 37,700 |
| Panel B: Severe Infant Health Outcome (<750 g, <26 weeks, or <5 Apgar) | | | | | |
| Coefficient $\beta_{1}$ | -0.0042 | -0.0017 | -0.0019 | -0.0007 | -0.0018 |
| Standard Error | 0.0002 | 0.0016 | 0.0017 | 0.0018 | 0.0025 |
| P-value | <0.0010 | 0.2850 | 0.2640 | 0.6970 | 0.4580 |
| Relative Change (%) | -23.7 | -9.9 | -10.7 | -3.9 | -10.4 |
| Observations | 41,620 | 41,620 | 41,620 | 38,515 | 37,700 |
| Panel C: Included Controls | | | | | |
| Year FE |  | X | X | X | X |
| County FE |  |  | X | X | X |
| Demographic Characteristics |  |  |  | X | X |
| All Remaining Controls |  |  |  |  | X |

Notes: Each coefficient is the policy's effect from a separate difference-in-differences model. Standard errors are clustered at the county level. The relative change is calculated as (coefficient ÷ treatment group’s pre-policy mean) × 100.

| Appendix Table A4: Outcomes of Pilot Program on White Married Mothers | | | | | |
| --- | --- | --- | --- | --- | --- |
| Panel A: Infant Mortality Rate Outcome | | | | | |
| Coefficient $\beta_{1}$ | -0.0015 | -0.0008 | -0.0008 | -0.0005 | -0.0016 |
| Standard Error | 0.00002 | 0.0003 | 0.0003 | 0.0003 | 0.0005 |
| P-value | <0.0010 | 0.0050 | 0.0080 | 0.0490 | 0.0010 |
| Relative Change (%) | -37.1 | -20.4 | -18.9 | -12.8 | -39.8 |
| Observations | 245,365 | 245,365 | 245,365 | 234,980 | 231,215 |
| Panel B: Severe Infant Health Outcome (<750 g, <26 weeks, or <5 Apgar) | | | | | |
| Coefficient $\beta_{1}$ | -0.0013 | -0.0013 | -0.0012 | -0.0011 | -0.0027 |
| Standard Error | 0.0001 | 0.0004 | 0.0004 | 0.0004 | 0.0005 |
| P-value | <0.0010 | 0.0020 | 0.0030 | 0.0080 | <0.0010 |
| Relative Change (%) | -22.7 | -23.7 | -22.0 | -19.6 | -48.0 |
| Observations | 245,365 | 245,365 | 245,365 | 234,980 | 231,215 |
| Panel C: Included Controls | | | | | |
| Year FE |  | X | X | X | X |
| County FE |  |  | X | X | X |
| Demographic Characteristics |  |  |  | X | X |
| All Remaining Controls |  |  |  |  | X |

Notes: Each coefficient is the policy's effect from a separate difference-in-differences model. Standard errors are clustered at the county level. The relative change is calculated as (coefficient ÷ treatment group’s pre-policy mean) × 100.

| Appendix Table A5: Sensitivity Analysis of High-Risk Combinations | | | |  |
| --- | --- | --- | --- | --- |
|  | Black Single Mothers | White Single  Mothers | Black Married  Mothers | White Married Mothers |
| Panel A: Severe Infant Health Outcome (<500 g, <28 weeks, or <4 Apgar) | | | | |
| Coefficient $\beta_{1}$ | -0.0070 | -0.0106 | -0.0004 | -0.0039 |
| Standard Error | 0.0016 | 0.0015 | 0.0025 | 0.0007 |
| P-value | <0.0010 | <0.0010 | 0.8810 | <0.0010 |
|  |  |  |  |  |
| Panel B: Severe Infant Health Outcome (<1000 g, <28 weeks, or <4 Apgar) | | | | |
| Coefficient $\beta_{1}$ | -0.0035 | -0.0102 | 0.0012 | -0.0036 |
| Standard Error | 0.0018 | 0.0017 | 0.0028 | 0.0008 |
| P-value | 0.0570 | <0.0010 | 0.6800 | <0.0010 |
|  |  |  |  |  |
| Panel C: Severe Infant Health Outcome (<500 g, <28 weeks, or <7 Apgar) | | | | |
| Coefficient $\beta_{1}$ | -0.0155 | -0.0156 | -0.0019 | -0.0026 |
| Standard Error | 0.0028 | 0.0028 | 0.0045 | 0.0011 |
| P-value | <0.0010 | <0.0010 | 0.6710 | 0.0270 |
|  |  |  |  |  |
| Panel D: Severe Infant Health Outcome (<1000 g, <28 weeks, or <7 Apgar) | | | | |
| Coefficient $\beta_{1}$ | -0.0128 | -0.0155 | -0.0016 | -0.0022 |
| Standard Error | 0.0028 | 0.0030 | 0.0047 | 0.0013 |
| P-value | <0.0010 | <0.0010 | 0.7370 | 0.0950 |
|  |  |  |  |  |
| Observations | 71,230 | 61,135 | 37,700 | 231,215 |
| Notes: Each coefficient is the policy's effect from a separate difference-in-differences model with county fixed effects, year fixed effects, month fixed effects, and all other explanatory variables. Standard errors are clustered at the county level. The relative change is calculated as (coefficient ÷ treatment group’s pre-policy mean) × 100. | | | | |

| Appendix Table A6: Outcomes of Pilot Program by Race & Marital Status by Age | | | | |
| --- | --- | --- | --- | --- |
|  | Black Single Mothers | White Single  Mothers | Black Married  Mothers | White Married Mothers |
| 1. Mother’s Under 30 Years Old | | | | |
| Panel A: Infant Mortality Rate Outcome | | | | |
| Coefficient $\beta_{1}$ | 0.0006 | -0.0017 | 0.0007 | -0.0036 |
| Standard Error | 0.0014 | 0.0009 | 0.0022 | 0.0006 |
| P-value | 0.6850 | 0.0790 | 0.7460 | <0.0010 |
| Treatment Pre-Policy Mean | 0.0110 | 0.0127 | 0.0096 | 0.0063 |
| Relative Change (%) | 5.2 | -13.1 | 7.4 | -58.0 |
| Observations | 56,300 | 53,555 | 16,350 | 124,870 |
|  |  |  |  |  |
| Panel B: Severe Infant Health Outcome (<750 g, <26 weeks, or <5 Apgar) | | | | |
| Coefficient $\beta_{1}$ | -0.0056 | -0.0108 | -0.0012 | -0.0042 |
| Standard Error | 0.0012 | 0.0016 | 0.0028 | 0.0006 |
| P-value | <0.0010 | <0.0010 | 0.6740 | <0.0010 |
| Treatment Pre-Policy Mean | 0.0214 | 0.0143 | 0.0135 | 0.0076 |
| Relative Change (%) | -26.4 | 75.7 | -8.8 | -54.8 |
| Observations | 56,300 | 53,555 | 16,350 | 124,870 |
| 1. Mother’s 30 Years and Older | | | | |
| Panel A: Infant Mortality Rate Outcome | | | | |
| Coefficient $\beta_{1}$ | -0.0007 | 0.0071 | 0.0076 | -0.0001 |
| Standard Error | 0.0032 | 0.0034 | 0.0021 | 0.0007 |
| P-value | 0.8210 | 0.0440 | 0.0010 | 0.9420 |
| Treatment Pre-Policy Mean | 0.0152 | 0.0072 | 0.0064 | 0.0025 |
| Relative Change (%) | -4.8 | 98.1 | 118.2 | -2.1 |
| Observations | 14,875 | 7,580 | 21,350 | 106,320 |
| Panel B: Severe Infant Health Outcome (<750 g, <26 weeks, or <5 Apgar) | | | | |
| Coefficient $\beta_{1}$ | -0.0204 | 0.0097 | -0.0018 | -0.0016 |
| Standard Error | 0.0032 | 0.0044 | 0.0045 | 0.0009 |
| P-value | <0.0010 | 0.0290 | 0.6850 | 0.0690 |
| Treatment Pre-Policy Mean | 0.0352 | 0.0144 | 0.0215 | 0.0041 |
| Relative Change (%) | -57.9 | 67.7 | -8.5 | -40.1 |
| Observations | 14,875 | 7,580 | 21,350 | 106,320 |
|  |  |  |  |  |
| Notes: Each coefficient is the policy's effect from a separate difference-in-differences model with county fixed effects, year fixed effects, month fixed effects, and all other explanatory variables. Standard errors are clustered at the county level. The relative change is calculated as (coefficient ÷ treatment group’s pre-policy mean) × 100. | | | | |

| Appendix Table A7: Outcomes of Pilot Program by Race & Marital Status by Education | | | | |
| --- | --- | --- | --- | --- |
|  | Black Single Mothers | White Single  Mothers | Black Married  Mothers | White Married Mothers |
| 1. Mother’s Education: High School Diploma or Less | | | | |
| Panel A: Infant Mortality Rate Outcome | | | | |
| Coefficient $\beta_{1}$ | 0.0006 | 0.0095 | 0.0074 | -0.0039 |
| Standard Error | 0.0016 | 0.0017 | 0.0039 | 0.0011 |
| P-value | 0.7220 | <0.0010 | 0.0630 | 0.0010 |
| Treatment Pre-Policy Mean | 0.0125 | 0.0070 | 0.0072 | 0.0079 |
| Relative Change (%) | 4.6 | 135.8 | 102.5 | -50.0 |
| Observations | 40,445 | 40,520 | 10,400 | 59,835 |
|  |  |  |  |  |
| Panel B: Severe Infant Health Outcome (<750 g, <26 weeks, or <5 Apgar) | | | | |
| Coefficient $\beta_{1}$ | -0.0065 | 0.0002 | -0.0017 | 0.0010 |
| Standard Error | 0.0019 | 0.0016 | 0.0044 | 0.0011 |
| P-value | 0.0010 | 0.9030 | 0.7040 | 0.3410 |
| Treatment Pre-Policy Mean | 0.0255 | 0.0140 | 0.0245 | 0.0059 |
| Relative Change (%) | -25.6 | 1.4 | -6.8 | 17.0 |
| Observations | 40,445 | 40,520 | 10,400 | 59,835 |
| 1. Mother’s Education: Some College or Higher | | | | |
| Panel A: Infant Mortality Rate Outcome | | | | |
| Coefficient $\beta_{1}$ | 0.0001 | -0.0125 | 0.0034 | -0.0013 |
| Standard Error | 0.0023 | 0.0020 | 0.0016 | 0.0006 |
| P-value | 0.9780 | <0.0010 | 0.0430 | 0.0260 |
| Treatment Pre-Policy Mean | 0.0105 | 0.0178 | 0.0082 | 0.0034 |
| Relative Change (%) | 0.6 | -70.2 | 41.6 | -39.0 |
| Observations | 30,730 | 20,615 | 27,300 | 171,355 |
| Panel B: Severe Infant Health Outcome (<750 g, <26 weeks, or <5 Apgar) | | | | |
| Coefficient $\beta_{1}$ | -0.0126 | -0.0189 | -0.0018 | -0.0037 |
| Standard Error | 0.0024 | 0.0031 | 0.0024 | 0.0005 |
| P-value | <0.0010 | <0.0010 | 0.4450 | <0.0010 |
| Treatment Pre-Policy Mean | 0.0211 | 0.0148 | 0.0153 | 0.0055 |
| Relative Change (%) | -60.0 | -127.8 | -11.9 | -67.0 |
| Observations | 30,730 | 20,615 | 27,300 | 171,355 |
|  |  |  |  |  |
| Notes: Each coefficient is the policy's effect from a separate difference-in-differences model with county fixed effects, year fixed effects, month fixed effects, and all other explanatory variables. Standard errors are clustered at the county level. The relative change is calculated as (coefficient ÷ treatment group’s pre-policy mean) × 100. | | | | |

| Appendix Table A8: Outcomes of Pilot Program by Race & Marital Status- Border Counties as Control Group | | | | |
| --- | --- | --- | --- | --- |
|  | Black Single Mothers | White Single  Mothers | Black Married  Mothers | White Married Mothers |
| Panel A: Infant Mortality Rate Outcome | | | | |
| Coefficient $\beta_{1}$ | 0.0014 | 0.0030 | 0.0064 | -0.0022 |
| Standard Error | 0.0021 | 0.0029 | 0.0031 | 0.0011 |
| P-value | 0.5270 | 0.3240 | 0.0690 | 0.0740 |
| Treatment Pre-Policy Mean | 0.0116 | 0.0115 | 0.0079 | 0.0041 |
| Relative Change (%) | 12.1 | 26.4 | 81.6 | -55.0 |
| Observations | 15,585 | 5,750 | 7,290 | 24,785 |
|  |  |  |  |  |
| Panel B: Severe Infant Health Outcome (<750 g, <26 weeks, or <5 Apgar) | | | | |
| Coefficient $\beta_{1}$ | -0.0044 | -0.0078 | -0.0037 | -0.0028 |
| Standard Error | 0.0051 | 0.0034 | 0.0043 | 0.0011 |
| P-value | 0.4060 | 0.0420 | 0.4060 | 0.0360 |
| Treatment Pre-Policy Mean | 0.0237 | 0.0141 | 0.0176 | 0.0055 |
| Relative Change (%) | -18.7 | -55.3 | -21.1 | -50.6 |
| Observations | 15,585 | 5,750 | 7,290 | 24,785 |
| Notes: Each coefficient is the policy's effect from a separate difference-in-differences model with county fixed effects, year fixed effects, month fixed effects, and all other explanatory variables. Standard errors are clustered at the county level. The relative change is calculated as (coefficient ÷ treatment group’s pre-policy mean) × 100. Border counties consist of Autauga, Barbour, Bullock, Crenshaw, Elmore, Lee, Lowndes, Pike and Tallapoosa. | | | | |

| Appendix Table A9: Robustness Checks for Infant Mortality | | | |  |
| --- | --- | --- | --- | --- |
|  | Black Single Mothers | White Single  Mothers | Black Married  Mothers | White Married Mothers |
| Panel A: Oster’s Bound | | | | |
| Beta when Delta= 1 | -315.8960 | -158.7170 | 32.4040 | -36.4030 |
| Beta when Delta= -1 | 0.0001 | 0.0030 | -0.0060 | 0.0100 |
|  |  |  |  |  |
| Panel B: Drop Covid-19 Lockdown Period (March 2020-April 2021) | | | | |
| Coefficient $\beta_{1}$ | -0.0005 | 0.0018 | 0.0036 | -0.0015 |
| Standard Error | 0.0016 | 0.0010 | 0.0020 | 0.0004 |
| P-value | 0.7750 | 0.0830 | 0.0740 | <0.0010 |
|  |  |  |  |  |
| Panel C: Use Year-Month Fixed Effects Instead of Year and Month Fixed Effects | | | | |
| Coefficient $\beta_{1}$ | -0.0013 | -0.0018 | 0.0044 | -0.0018 |
| Standard Error | 0.0016 | 0.0012 | 0.0018 | 0.0006 |
| P-value | 0.4080 | 0.1380 | 0.0150 | 0.0020 |
|  |  |  |  |  |
| Panel D: Logit Model | | | | |
| Coefficient $\beta_{1}$ | -0.0732 | 0.0004 | 0.5302 | -0.3811 |
| Standard Error | 0.1692 | 0.1817 | 0.2336 | 0.1529 |
| P-value | 0.6650 | 0.9980 | 0.0230 | 0.0130 |
|  |  |  |  |  |
| Panel E: Probit Model | | | | |
| Coefficient $\beta_{1}$ | -0.0059 | 0.0400 | 0.1802 | -0.1867 |
| Standard Error | 0.0651 | 0.0688 | 0.0885 | 0.0520 |
| P-value | 0.9270 | 0.5610 | 0.0420 | <0.0010 |
|  |  |  |  |  |
| Observations | 71,230 | 61,135 | 37,700 | 231,215 |
| Notes: Each coefficient is the policy's effect from a separate difference-in-differences model with county fixed effects, year fixed effects, month fixed effects (except Panel C), and all other explanatory variables. Standard errors are clustered at the county level. The relative change is calculated as (coefficient ÷ treatment group’s pre-policy mean) × 100. | | | | |

| Appendix Table A10: Robustness Checks for Severe Infant Health Outcome (<750 g, <26 weeks, or <5 Apgar) | | | | |
| --- | --- | --- | --- | --- |
|  | Black Single Mothers | White Single  Mothers | Black Married  Mothers | White Married Mothers |
| Panel A: Oster’s Bound | | | | |
| Beta when Delta= 1 | 266.1180 | 0.00740 | 55.4140 | -16.8410 |
| Beta when Delta= -1 | -0.0100 | -0.0250 | -0.0070 | 0.0170 |
|  |  |  |  |  |
| Panel B: Drop Covid-19 Lockdown Period (March 2020-April 2021) | | | | |
| Coefficient $\beta_{1}$ | -0.0105 | -0.0087 | 0.0004 | -0.0026 |
| Standard Error | 0.0015 | 0.0021 | 0.0025 | 0.0005 |
| P-value | <0.0010 | <0.0010 | 0.8660 | <0.0010 |
|  |  |  |  |  |
| Panel C: Use Year-Month Fixed Effects Instead of Year and Month Fixed Effects | | | | |
| Coefficient $\beta_{1}$ | -0.0093 | -0.0070 | -0.0013 | -0.0031 |
| Standard Error | 0.0015 | 0.0016 | 0.0023 | 0.0005 |
| P-value | <0.0010 | <0.0010 | 0.5650 | <0.0010 |
|  |  |  |  |  |
| Panel D: Logit Model | | | | |
| Coefficient $\beta_{1}$ | -0.5963 | -0.7497 | -0.0878 | -0.3660 |
| Standard Error | 0.0773 | 0.1616 | 0.1675 | 0.0836 |
| P-value | <0.0010 | <0.0010 | 0.6000 | <0.0010 |
|  |  |  |  |  |
| Panel E: Probit Model | | | | |
| Coefficient $\beta_{1}$ | -0.2917 | -0.3644 | -0.0850 | -0.1424 |
| Standard Error | 0.0425 | 0.0692 | 0.0768 | 0.0309 |
| P-value | <0.0010 | <0.0010 | 0.2680 | <0.0010 |
|  |  |  |  |  |
| Observations | 71,230 | 61,135 | 37,700 | 231,215 |
| Notes: Each coefficient is the policy's effect from a separate difference-in-differences model with county fixed effects, year fixed effects, month fixed effects (except Panel C), and all other explanatory variables. Standard errors are clustered at the county level. The relative change is calculated as (coefficient ÷ treatment group’s pre-policy mean) × 100. | | | | |

| Appendix Table A11: Synthetic Control Counties | | |  |
| --- | --- | --- | --- |
| Black Single Mothers | | White Single Mothers | |
| Counties | Weight | Counties | Weight |
| Autauga | 0.001 | Colbert | 0.198 |
| Blount | 0.010 | Greene | 0.169 |
| Bullock | 0.242 | Henry | 0.345 |
| Cherokee | 0.011 | Jefferson | 0.284 |
| Dallas | 0.075 | Marengo | 0.004 |
| Geneva | 0.034 |  |  |
| Henry | 0.074 |  |  |
| Madison | 0.351 |  |  |
| Mobile | 0.065 |  |  |
| Pike | 0.095 |  |  |
| Tallapoosa | 0.044 |  |  |
| RMSPE | 0.00176 | RMSPE | 0.01027 |
|  |  |  |  |
| Black Married Mothers | | White Married Mothers | |
| Counties | Weight | Counties | Weight |
| Bullock | 0.016 | Butler | 0.031 |
| Dallas | 0.082 | Conecuh | 0.016 |
| DeKalb | 0.022 | Geneva | 0.024 |
| Geneva | 0.010 | Greene | 0.180 |
| Dallas | 0.075 | Jefferson | 0.694 |
| Geneva | 0.034 | Wilcox | 0.054 |
| Lamar | 0.001 |  |  |
| Lee | 0.220 |  |  |
| Lowndes | 0.001 |  |  |
| Madison | 0.096 |  |  |
| Marengo | 0.001 |  |  |
| Perry | 0.052 |  |  |
| Pike | 0.211 |  |  |
| Randolph | 0.015 |  |  |
| St. Clair | 0.274 |  |  |
| RMSPE | 0.00045 | RMSPE | 0.00045 |
| Note: All other counties in Alabama received a weight of zero, except for Macon, Montgomery and Russell which were excluded due to their connection with the pilot program. Choctaw is also excluded due to missing data. RMSPE stands for Root Mean Squared Prediction Error. | | | |

| Appendix Table A12: Estimated Treatment Effect from Synthetic Control Method | | | | |
| --- | --- | --- | --- | --- |
| Year | Black Single Mothers | White Single  Mothers | Black Married  Mothers | White Married Mothers |
| Average | 0.0001 | -0.0055 | 0.0035 | -0.0010 |
| 2019 | 0.0018 | -0.0044 | -0.0006 | -0.0005 |
| 2020 | 0.0007 | -0.0137 | 0.0010 | -0.0001 |
| 2021 | -0.0001 | -0.0037 | 0.0029 | -0.0008 |
| 2022 | 0.0045 | -0.0170 | 0.0056 | -0.0018 |
| 2023 | -0.0064 | 0.0112 | 0.0088 | -0.0018 |
| Notes: Each estimate, α*_1t_* from equation (2) is the difference in infant mortality between the treated group and the synthetic group. Each column is from a separate synthetic control model which can be visually seen in Figure 2. | | | | |

| Appendix Table A13: Delivery Payment & Supplemental Program Outcomes from Pilot Program | | | | |
| --- | --- | --- | --- | --- |
|  | Black Single Mothers | White Single  Mothers | Black Married  Mothers | White Married Mothers |
| Panel A: Private Insurance Outcome | | | | |
| Coefficient $\beta_{1}$ | 0.0546 | 0.0864 | 0.0337 | 0.0132 |
| Standard Error | 0.0152 | 0.0189 | 0.0196 | 0.0163 |
| P-value | 0.0010 | <0.0010 | 0.0910 | 0.4230 |
|  |  |  |  |  |
| Panel B: Medicaid Outcome | | | | |
| Coefficient $\beta_{1}$ | -0.0650 | -0.0856 | -0.0391 | -0.0227 |
| Standard Error | 0.0167 | 0.0191 | 0.0208 | 0.0186 |
| P-value | <0.0010 | <0.0010 | 0.0650 | 0.2270 |
|  |  |  |  |  |
| Panel C: Women, Infants, and Children (WIC) Supplemental Nutrition Program Outcome | | | | |
| Coefficient $\beta_{1}$ | -0.0397 | -0.0076 | -0.0045 | 0.0250 |
| Standard Error | 0.0207 | 0.0283 | 0.0244 | 0.0169 |
| P-value | 0.0600 | 0.7890 | 0.8530 | 0.1450 |
|  |  |  |  |  |
| Observations | 71,230 | 61,135 | 37,700 | 231,215 |
| Notes: Each coefficient is the policy's effect from a separate difference-in-differences model with county fixed effects, year fixed effects, month fixed effects, and all other explanatory variables. Standard errors are clustered at the county level. The relative change is calculated as (coefficient ÷ treatment group’s pre-policy mean) × 100. | | | | |

Appendix Figures A1: Treated vs Control Counties by Race and Marital Status

Appendix Figures A2: Sensitivity Analysis of Parallel Trends assumption by implementing the methodology proposed by Rambachan and Roth

Figure A2.a) Black Single Mothers Figure A2.b) White Single Mothers


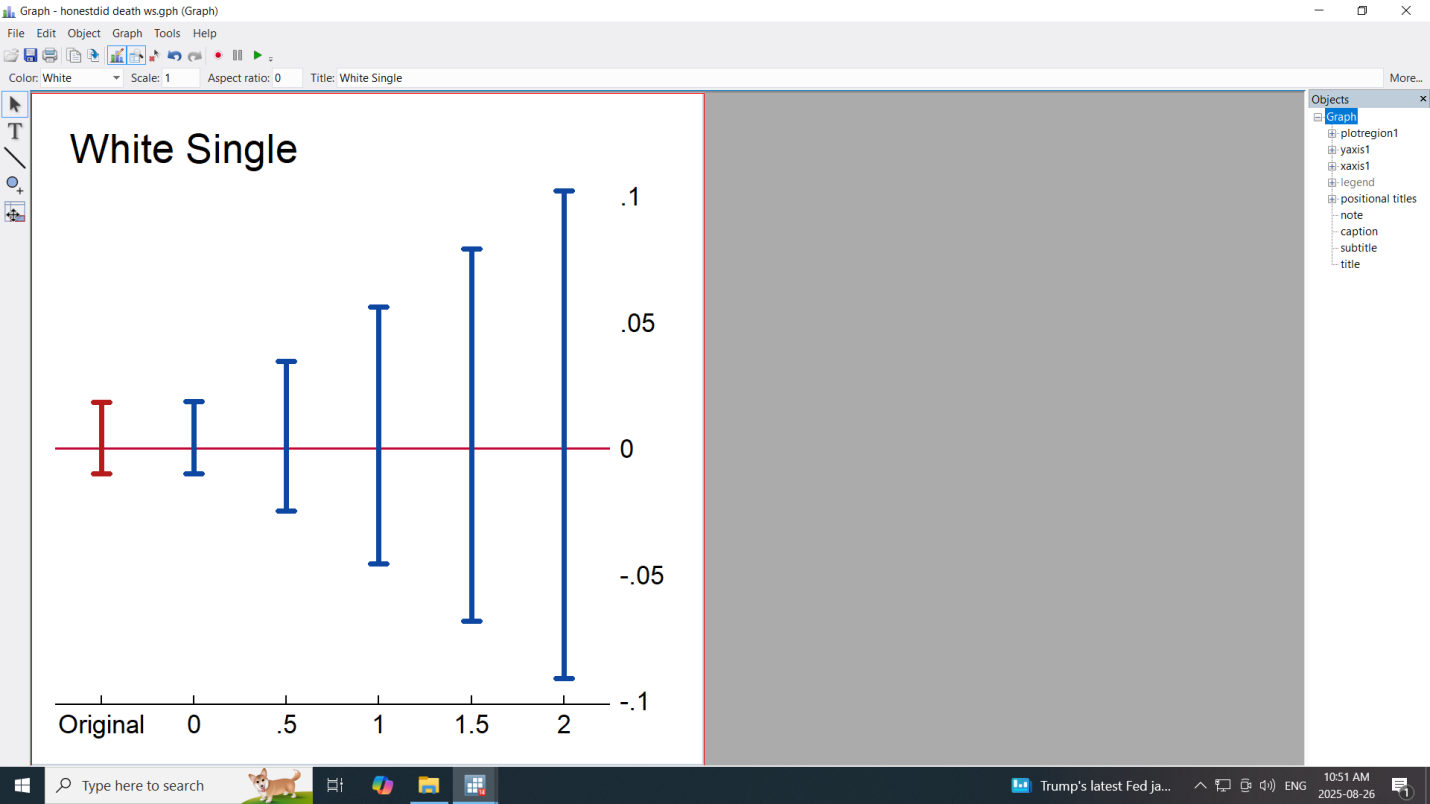

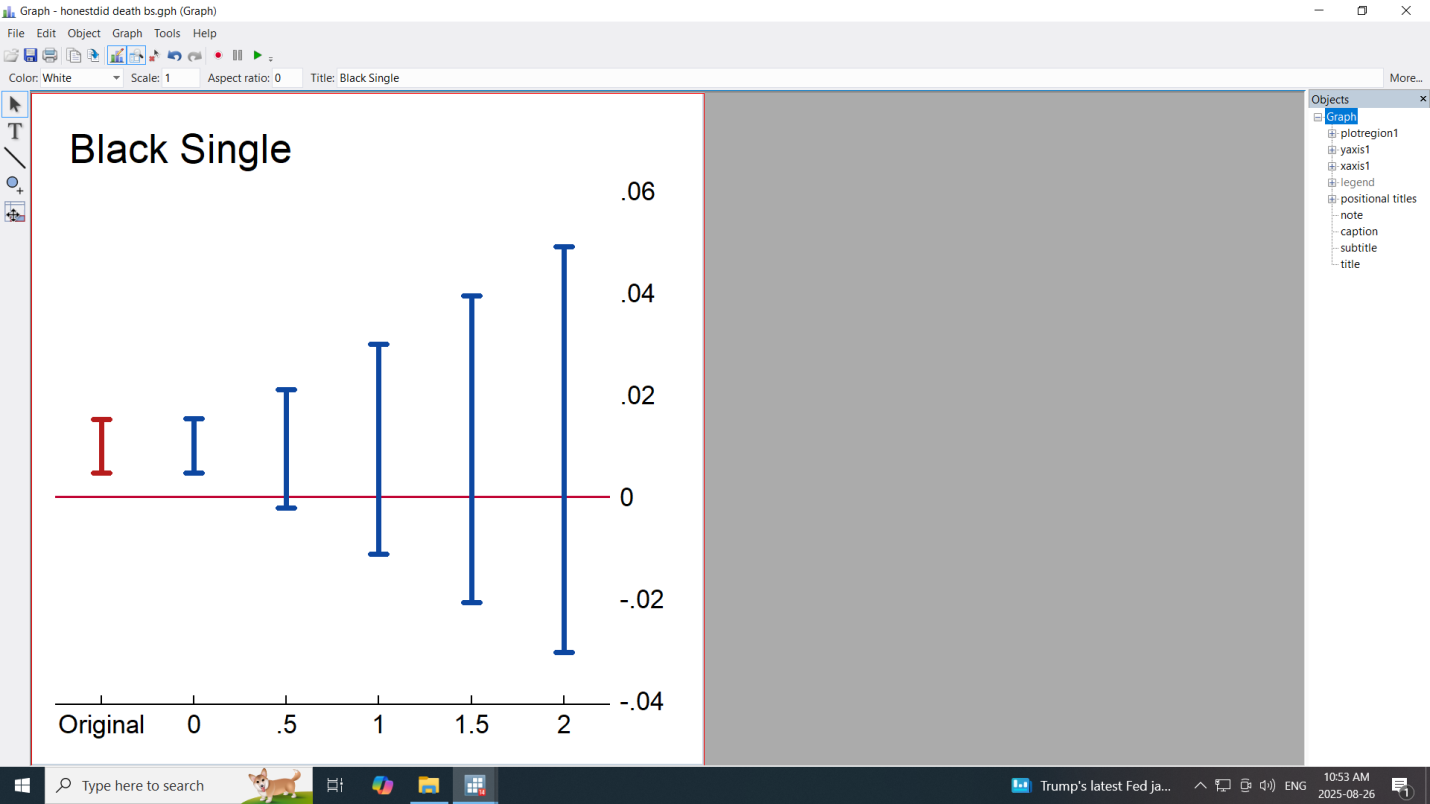


Figure A2.c) Black Married Mothers Figure A2.d) White Married Mothers


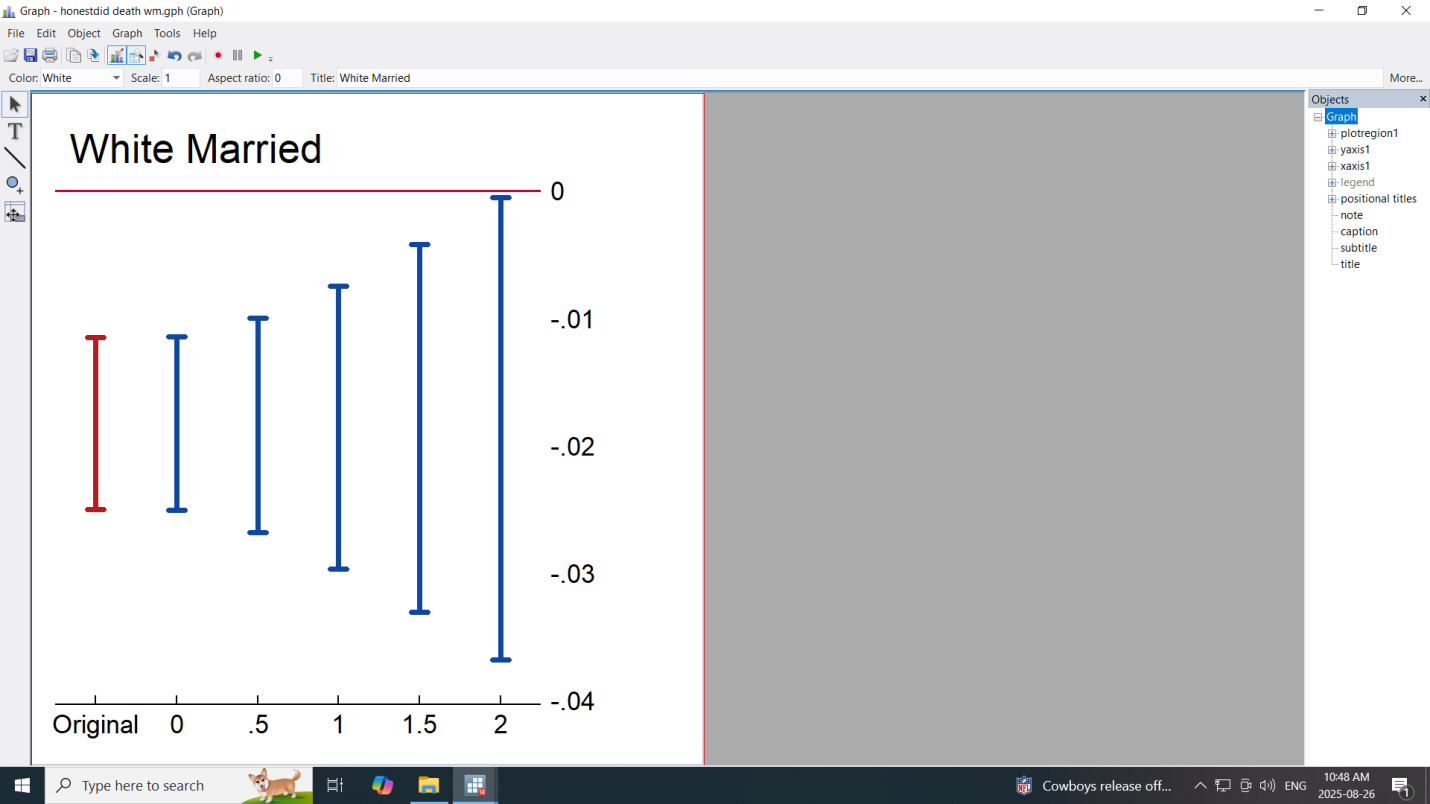

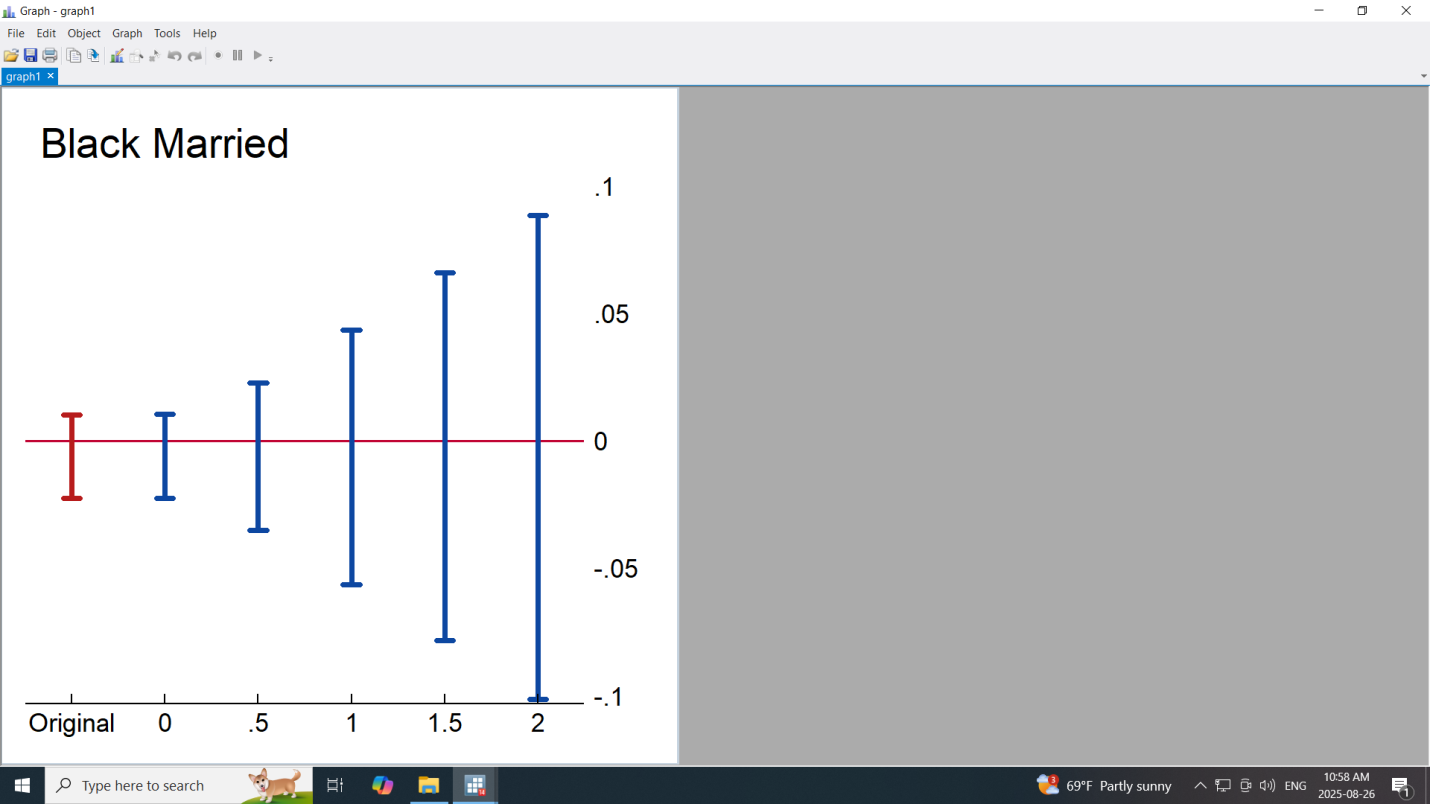


Note: Using the methodology proposed by Rambachan and Roth (2022) we check the sensitivity of the parallel trends assumption on our event-studies in Figure 1. We use the honestdid Stata package.
